# Efficient and rapid isolation of native AMPA receptor complexes for cryo‐EM

**DOI:** 10.1002/pro.70483

**Published:** 2026-01-24

**Authors:** Jumi Park, Eric Gouaux

**Affiliations:** ^1^ Vollum Institute Oregon Health & Science University Portland Oregon USA; ^2^ Howard Hughes Medical Institute Oregon Health & Science University Portland Oregon USA

**Keywords:** AMPA receptor, cryo‐EM, native membrane proteins, rapid isolation

## Abstract

Isolating native ion channels for structural characterization is routinely achieved by extraction from membrane fractions of tissue with prolonged mild detergent treatment. AMPA receptors (AMPARs), glutamatergic receptors that mediate fast excitatory transmission and synaptic plasticity, are coassembled with diverse auxiliary subunits and transiently‐interacting partners to finely regulate processes from trafficking to gating kinetics. Previous studies of the composition and architecture of native AMPARs (nAMPARs) isolated from membrane fractions of rodent brain tissue have revealed many different subunit compositions and non‐stochastic assemblies of the auxiliary subunits. However, elucidating the molecular architectures of nAMPARs complexed with less populated or transiently bound proteins has proven challenging. Here, we employ strategies for the rapid solubilization and purification of nAMPARs to increase the likelihood of isolating the greatest range of nAMPARs complexes. By utilizing whole brain tissue and reducing solubilization and purification duration, we purify nAMPARs complexed with a wider variety of auxiliary subunits and binding partners in a sufficient quantity and purity for cryo‐electron microscopy studies. We resolve previously unreported subunit compositions and conformations that include ones with a half‐splayed ATD layer, as well as complexes with four distinct auxiliary subunit arrangements in the TMD layer.

## 1 INTRODUCTION

Ion channels in the central nervous system (CNS) exhibit functional diversity and regulation through heteromeric pore‐forming subunits and association with a broad range of auxiliary subunits. Along with technical progress in single‐particle cryogenic electron microscopy (cryo‐EM), recent advances in structural studies of ion channels in CNS have been achieved by isolating native ion channels from CNS tissue and elucidating their high‐resolution structures, thus providing new insights into their physiologically relevant heteromeric assemblies (Sun et al., 2023; Zhang et al., 2025; Zhao et al., 2019; Zhou et al., 2025; Zhu & Gouaux, 2021). Structural analyses of native ion channels such as glycine receptors from spinal cord and brainstem (Zhu & Gouaux, 2021) and NMDA receptors from cerebral cortex and hippocampus (Zhang et al., 2025) have revealed heteromeric receptor assemblies and stoichiometry, as well as assembly intermediates. Moreover, the pharmacology and association with auxiliary subunits and binding proteins of ion channels have been successfully elucidated with GABA_A_ receptors from mouse brain (Sun et al., 2023) and most recently from the human brain of epileptic patients (Zhou et al., 2025). Among these, AMPA receptors (AMPARs) have been studied extensively (Fang et al., 2025; Yu et al., 2021; Zhao et al., 2019) as they play crucial roles in fast excitatory transmission and synaptic plasticity in the brain (Diering & Huganir, 2018; Malinow & Malenka, 2002) where various auxiliary subunits and transiently binding partners modulate channel gating, ion permeability, pharmacological properties, as well as trafficking, thereby sculpting synapse development, function and plasticity (Matthews et al., 2021). Moreover, the molecular compositions of AMPARs exhibit significant heterogeneity across different neuronal cell types, brain regions and even individual synapses (Geiger et al., 1995; Schwenk et al., 2014). This diversity, driven by the distinct expression of different receptors and auxiliary subunits, allows the functional properties of AMPARs to be precisely tailored to the specific physiological conditions of these local environments.

Structural and biophysical studies over the past 3 decades have revealed that AMPARs are tetrameric assemblies composed of GluA1 to GluA4 receptor subunits, which typically form a modular “Y‐shaped” conformation in the resting, non‐desensitized state. The receptor assembly has three well defined layers: the N‐terminal domain (ATD or NTD), the ligand‐binding domain (LBD), and the transmembrane domain (TMD) (Sobolevsky et al., 2009). In addition, transmembrane auxiliary subunits such as transmembrane AMPA receptor regulatory proteins (TARPs), germ cell‐specific gene 1‐like (GSG1L), and cornichon homolog‐2 and ‐3 (CNIH2/3) interact primarily with the TMD, the LBD–TMD linkers and, in select instances, the LBD, thus positioned to modulate nearly all aspects of receptor function (Chen et al., 2017; Gangwar et al., 2023; Herguedas et al., 2019; Klykov et al., 2021; Nakagawa, 2019; Pokharna et al., 2025; Twomey et al., 2016, 2017; Vega‐Gutiérrez et al., 2025; Zhang et al., 2021; Zhang, Ivica, et al., 2023; Zhang, Lape, et al., 2023; Zhao et al., 2016). Furthermore, recent studies of native AMPARs (nAMPARs) from rodent brain tissue have uncovered previously unseen subunit compositions of different AMPAR subtypes, constellations of TMD‐binding auxiliary subunits, and an ATD‐binding protein, derived from different brain regions (Fang et al., 2025; Yu et al., 2021; Zhao et al., 2019). However, given that there is a substantial group of proteins that participate in AMPAR assembly, trafficking, and function (Chen et al., 2014; Schwenk et al., 2012; Schwenk et al., 2014; Schwenk et al., 2019), further studies are required to understand the complete ensemble of AMPAR assemblies in vivo, from biogenesis to synaptic localization and internalization.

Isolation of nAMPARs typically has been achieved through detergent treatment of crude membrane fractions derived from rodent brain tissue (Yu et al., 2021; Zhao et al., 2019). This solubilization and purification strategy has yielded nAMPARs associated with postsynaptic density protein 95 (PSD‐95) (Zhao et al., 2019) and TARP *γ*8, CNIH2, and SynDIG4, also known as proline‐rich transmembrane protein 1 (PRRT1) (Yu et al., 2021). In addition, other transmembrane, secreted, or cytoplasmic proteins that transiently interact with AMPARs were co‐purified with nAMPARs from membrane fractions of rodent brain tissues under mild detergent conditions for proteomic analyses (Schwenk et al., 2012). Fractionation of crude membranes helps to reduce the possibility of contaminants and impurities by enriching membrane proteins of interest, which is advantageous for subsequent purification of receptor complexes.

**FIGURE 1:**
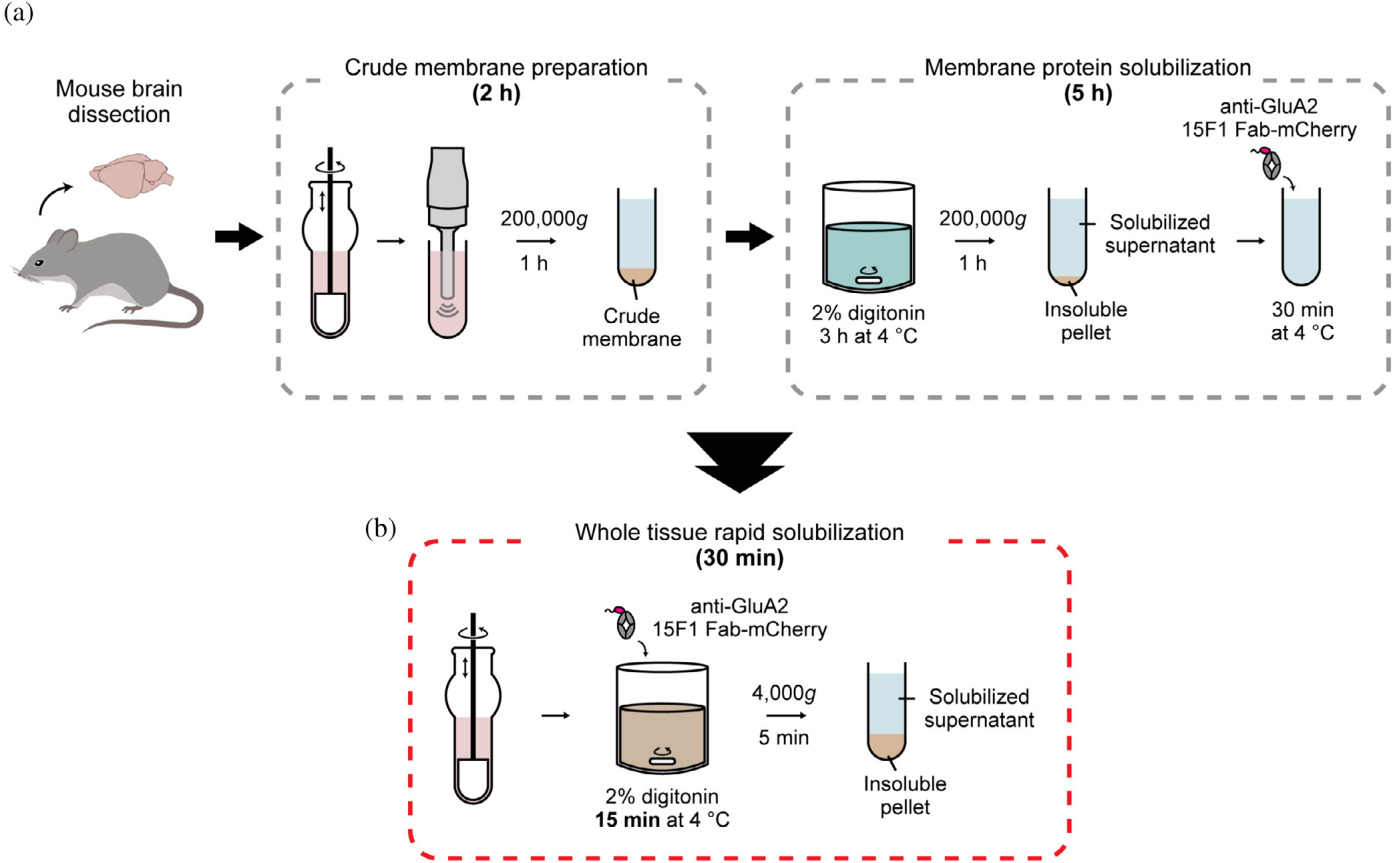
Strategy for rapid purification of nAMPARs from mouse brain. (a) Conventional workflow for extraction of nAMPARs from rodent brain membranes, involving a two‐step process of crude membrane preparation followed by membrane protein solubilization. The entire procedure requires approximately 7 h from brain dissection to generation of solubilized supernatant labeled with affinity‐tagged antibody. (b) Simplified procedure for extracting nAMPARs complexes by directly solubilizing dissected whole brain tissues with a short incubation period (15 min). Fluorescence‐ and affinity‐tagged anti‐GluA2 15F1 Fab are included during solubilization step, enabling preparation of affinity‐ready solubilized supernatant within 30 min.

The traditional approaches to the preparation of an affinity chromatography‐ready sample for nAMPARs from rodent brain have been accomplished through a multi‐hour, two‐step process (Yu et al., 2021; Zhao et al., 2019) (Figure 1a). First, crude membrane fractions are typically prepared by ultracentrifugation of homogenized brain tissue and then solubilized by detergent for an extended period, followed by another round of ultracentrifugation to remove insoluble materials. Consequently, it requires 6–7 h to obtain a purification‐ready solubilized protein sample from dissected brain. Subsequently, purification of solubilized nAMPARs has been performed by employing gravity‐flow immunoaffinity chromatography combined with elution by a displacing agent after incubating the solubilized supernatant with affinity‐tagged antibody fragments, requiring another 4–5 h to obtain purified nAMPARs, which includes size‐exclusion chromatography (SEC). Therefore, the entire process from dissection to purification usually takes ~12 h, or as long as 2 days for large‐scale experiments. However, considering that some interacting partners may form transient or weak interactions (Hanley, 2018; Nair et al., 2013; Opazo & Choquet, 2011; Suzuki et al., 2020), prolonged sample preparation such as separation of soluble and membrane fractions, extended solubilization, and gravity‐based purification may result in the dissociation of these complexes, thus limiting one's ability to isolate and ultimately understand their functional and molecular mechanisms. To address this limitation of the traditional isolation approach, we have developed a rapid isolation strategy based on short time‐scale solubilization of nAMPARs from brain tissue, rapid purification using magnetic beads, brief and efficient protease cleavage, and HPLC‐based fluorescence‐detection SEC (FSEC) (Kawate & Gouaux, 2006), thus enabling the preparation of purified nAMPAR complexes within 2 h.

## 2 RESULTS

### 2.1 Streamlined solubilization of nAMPARs from whole brain tissue

To reduce the time of sample preparation, we have made several key modifications to the process (Figure 1b). First, we omit membrane fractionation and instead solubilize whole brain tissue with detergent immediately after homogenization. In addition, we assess the extent to which we can reduce the duration of detergent incubation and thus preserve transiently associated proteins. Finally, we replace ultracentrifugation with a brief low‐speed centrifugation followed by filtration to clarify the solubilized supernatant, thereby reducing the total processing time to 30 min.

**FIGURE 2:**
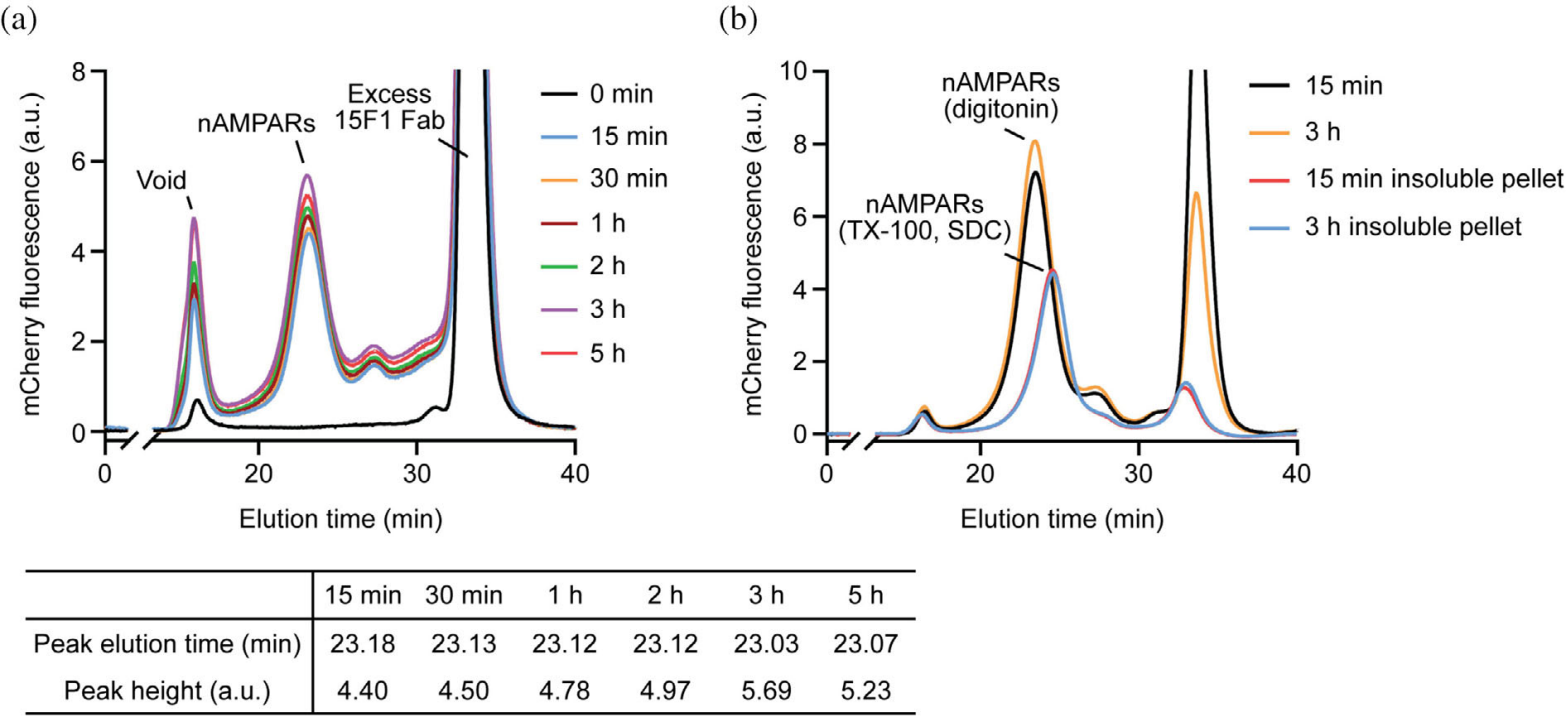
FSEC analyses of time‐dependent solubilization of nAMPARs. (a) FSEC profiles of solubilized supernatants from whole brain tissue extracted with 2% (w/v) digitonin for varying incubation times, detected by mCherry fluorescence of anti‐GluA2 15F1 Fab included in the supernatants. The elution time and peak height of nAMPAR at each time point are summarized in the lower table. (b) FSEC profiles of re‐solubilized supernatants by Triton X‐100 (TX‐100) and sodium deoxycholate (SDC) from the insoluble pellets obtained after 15 min or 3 h digitonin solubilization. mCherry fluorescence of anti‐GluA2 15F1 Fab was used for detection. a.u., arbitrary units.

To evaluate whether the detergent incubation time could be reduced without significantly affecting the solubilization efficiency, whole brain tissues were solubilized for various incubation times ranging from 15 min to 5 h. The solubilized supernatants from each time point were clarified by low‐speed centrifugation, followed by filtration, and then incubated with the anti‐GluA2 15F1 Fab containing a C‐terminal mCherry fluorescent tag. The solubilization efficiency of nAMPARs was analyzed using FSEC. The resulting FSEC profiles revealed that the yield of solubilized nAMPARs was maximal when the detergent incubation time was 3 h (Figure 2a). Nevertheless, a 15 min incubation achieved 80% solubilization efficiency relative to the 3 h time point, indicating acceptable performance within a significantly shorter time frame. Furthermore, when the amount of nAMPARs trapped in insoluble fractions after solubilization was assessed by resolubilizing the pellet from low‐speed centrifugation under a harsh detergent condition of 1% Triton X‐100 and 0.5% sodium deoxycholate (Li et al., 2010) and analyzed by FSEC (Figure 2b), the amount of non‐solubilized nAMPARs was nearly identical between the 15 min and 3 h conditions, suggesting that a 15 min detergent incubation is sufficient to extract the majority of soluble nAMPARs from whole brain tissue.

### 2.2 Magnetic beads and on‐resin protease cleavage accelerate efficient immunoaffinity purification

**FIGURE 3:**
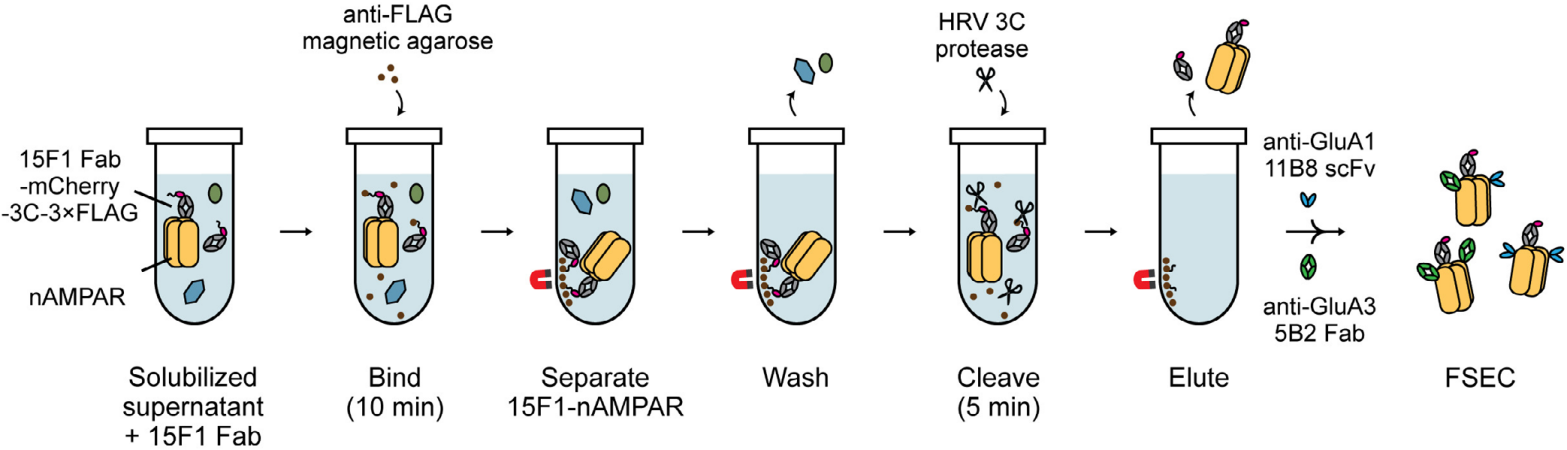
Workflow for purification of nAMPARs using anti‐GluA2 15F1 Fab‐mCherry‐3C‐3 × FLAG and anti‐FLAG magnetic resin with HRV 3C protease cleavage. Solubilized nAMPARs are captured on anti‐FLAG magnetic resin via 3C‐3 × FLAG‐tagged anti‐GluA2 15F1 Fab‐mCherry. The resin is rapidly separated from the flow‐through by a magnet and subsequently washed. HRV 3C protease treatment enables rapid and specific cleavage at the 3C recognition site within 15F1 Fab, resulting in the elution of 15F1 Fab‐nAMPAR complexes from the resin. The eluted complexes are subsequently incubated with subunit‐specific antibody fragments and further purified by FSEC.

Previous studies have employed gravity‐flow chromatography for the purification of nAMPARs, in combination with displacing agents to competitively elute the antibody‐nAMPAR complexes from the resin (Yu et al., 2021; Zhao et al., 2019). To most rapidly isolate nAMPARs solubilized from whole brain tissue, we modified the previously employed immunoaffinity purification strategy that utilized the anti‐GluA2 15F1 Fab‐mCherry construct configured with an HRV 3C protease recognition site and 3 × FLAG tag at the C‐terminus (Figure 3). First, we opted to use anti‐FLAG magnetic agarose beads for rapid and convenient purification because the high affinity and antigen–antibody specificity of the FLAG system enable the specific isolation of FLAG‐tagged proteins with minimal background. Furthermore, the magnetic bead format facilitated rapid yet gentle separation and washing of the resin, thereby minimizing total handling and purification time to preserve labile protein complexes. Next, target proteins were eluted by on‐resin protease cleavage, which enables highly specific elution with minimal contaminants. Here, we employed HRV 3C protease due to its high specificity and activity at 4°C. To further shorten the overall time of purification and elution, we employed a high concentration of protease (threefold excess relative to 15F1 Fab), which enabled a brief incubation of 5 min to achieve full cleavage. Cleaved and eluted proteins were separated from the magnetic beads and further incubated with anti‐GluA1 11B8 scFv and anti‐GluA3 5B2 Fab to label the corresponding receptor subunits. Antibody‐labeled nAMPARs were then purified by FSEC, utilizing an HPLC, to remove excess antibodies and proteases, and to isolate a monodisperse receptor complex.

**FIGURE 4:**
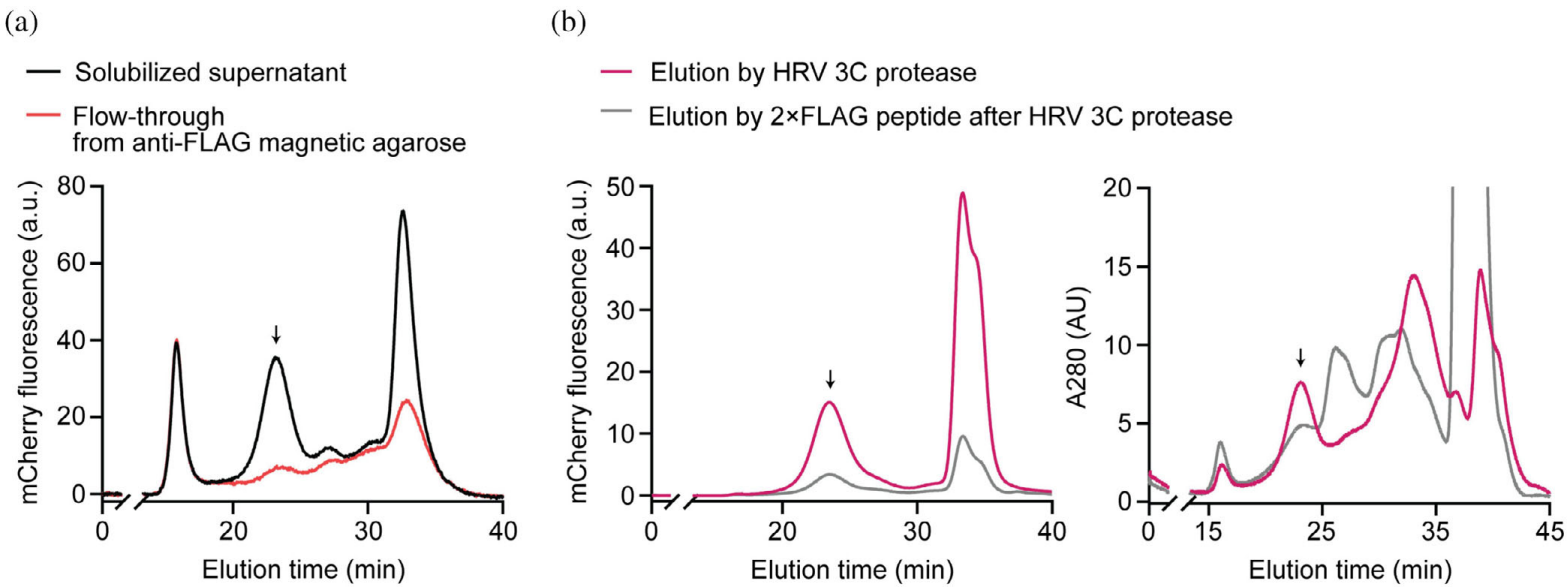
FSEC analyses of purification within 30 min of nAMPARs using anti‐FLAG magnetic resin and protease cleavage. (a) FSEC traces of the solubilized supernatant (black) and the flow‐through collected after 10 min incubation with anti‐FLAG magnetic resin (red), analyzed by mCherry fluorescence of 15F1 Fab. (b) FSEC traces of materials eluted by a 5 min treatment with HRV 3C protease (magenta) and subsequently eluted with 2 × FLAG peptide (gray), analyzed by mCherry fluorescence of 15F1 Fab (left) and absorbance at 280 nm (right). a.u., arbitrary units; AU, absorbance.

FSEC analyses of each purification step, where we traced the mCherry fluorescent signal derived from the 15F1 Fab, demonstrated that nAMPARs were rapidly isolated within 30 min by this modified immunoaffinity purification strategy (Figure 4). Most nAMPARs rapidly solubilized from whole brain tissue were efficiently captured by anti‐FLAG magnetic beads via 3 × FLAG‐tagged 15F1 Fab, thus were depleted in the flow‐through (Figure 4a). Next, the majority of captured nAMPARs were successfully eluted by protease cleavage with high purity, as evaluated by a single monodisperse peak at the expected elution time of nAMPARs from the mCherry fluorescence and absorbance at 280 nm (Figure 4b). Subsequent elution by 2 × FLAG peptide after protease cleavage showed few nAMPARs remaining on the beads, confirming efficient elution by protease. In summary, this rapid purification strategy combines a short solubilization time and magnetic bead‐based affinity capture followed by protease cleavage to enable efficient, high‐yield, and high‐purity isolation of nAMPARs.

### 2.3 Preservation of nAMPAR complexes by rapid isolation

**FIGURE 5:**
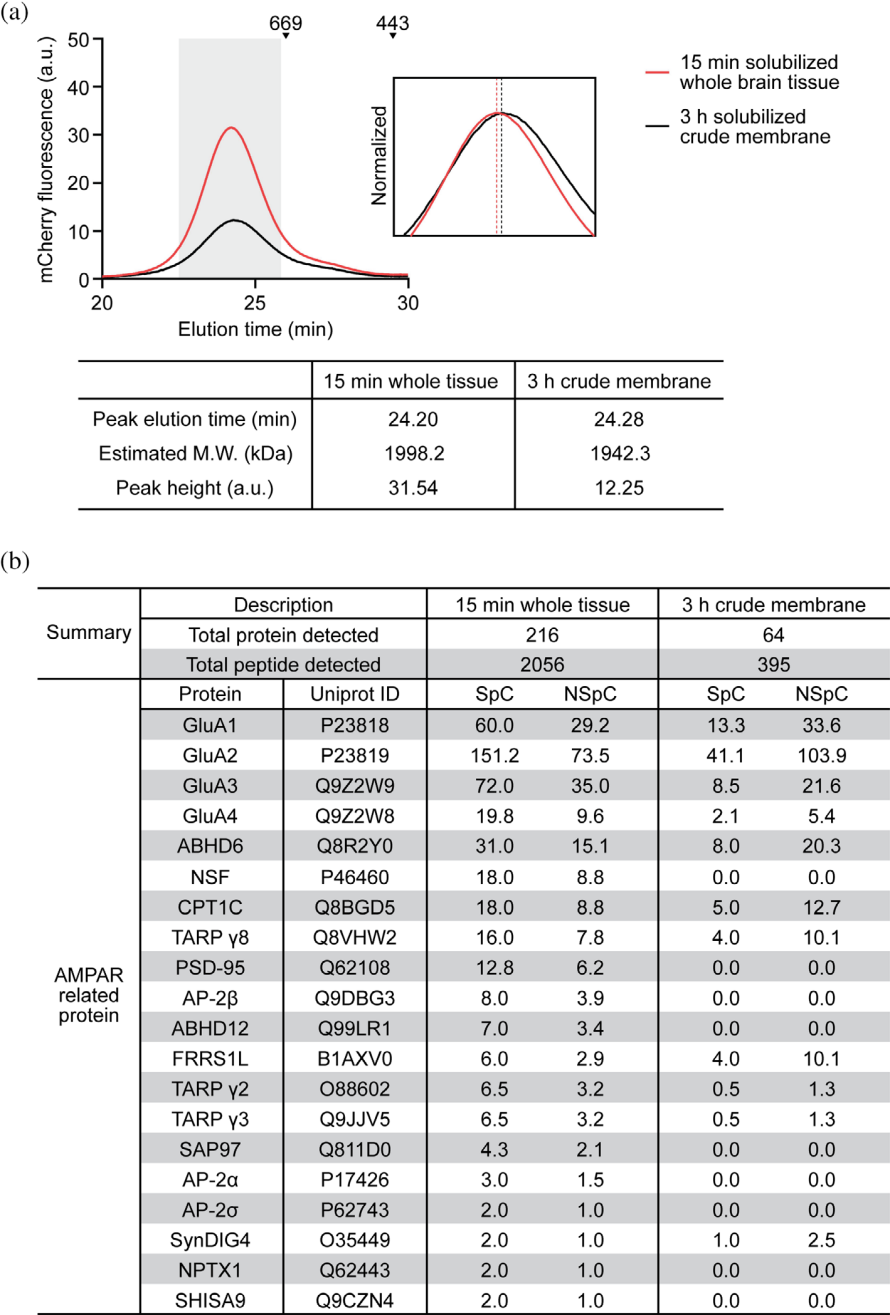
Rapid solubilization and purification captures AMPAR assemblies with higher yield. (a) FSEC traces of purified nAMPARs solubilized for 15 min from whole brain tissue (red) or for 3 h from crude membrane (black), analyzed by mCherry fluorescence of 15F1 Fab. The inset represents normalized FSEC traces within the highlighted range of elution time and dashed lines indicate peak elution time of each trace. The table below shows peak elution time, estimated molecular weight (M.W.), and peak height of each method. a.u., arbitrary units. (b) Mass spectrometry analysis of the front half of nAMPAR peak from each method. Proteins detected with at least two counts were included in the total protein detected. Spectral count (SpC) of each protein detected in each method is normalized by scaling total SpC to 1000 (NSpC, normalized SpC).

To evaluate the efficiency of the rapid isolation strategy for nAMPARs, we compared nAMPARs purified by rapid solubilization of whole brain tissue with those purified by prolonged solubilization of crude membranes prepared from the same mice. In both cases, purification of nAMPARs was carried out using the modified rapid purification with magnetic beads and protease‐mediated elution. The nAMPARs rapidly isolated from whole brain tissue eluted slightly earlier in the FSEC profile than those from crude membranes with long solubilization, corresponding to an estimated ~50 kDa increase in molecular weight of nAMPAR complexes (Figure 5a). In addition to the size difference, the overall yield of nAMPARs rapidly purified from whole brain tissue was approximately 2.6‐fold higher compared to that from crude membrane. Thus, the rapid isolation procedure not only reduces the time required for receptor isolation, but it also boosts the overall yield.

We further analyzed the leading half of the FSEC peak from each method by mass spectrometry. This fraction was collected to preferentially enrich for nAMPARs associated with the largest, and presumably most “intact” complexes, as shown by the profiles observed in Figure 5a. By focusing on these fractions, we aimed to assess whether protein compositions of nAMPARs rapidly isolated from whole brain preserved a more diverse and comprehensive set of interacting proteins (Figure 5b). The results revealed that over threefold more total proteins and fivefold more total peptides were detected in the sample derived from rapid solubilization of whole brain tissue compared to the traditional method. This indicates that protein samples from rapid solubilization are ostensibly less pure because they contain cytosolic proteins such as molecular chaperones and vesicle trafficking machinery as well as synaptic proteins such as cell adhesion molecules, synaptic vesicle fusion machinery, and postsynaptic scaffolding proteins. Nonetheless, higher spectral peptide counts for core subunits of nAMPARs, along with many previously identified auxiliary subunits such as ABHD6, CPT1C, and TARP *γ*8 were observed in the sample obtained via rapid solubilization of whole brain tissue, consistent with a higher yield of rapidly isolated nAMPARs, analyzed by FSEC (Figure 5a).

The rapid isolation method enabled co‐purification of several known nAMPAR‐associated proteins that were absent in the traditional crude membrane approach (Figure 5b). In addition to type I TARP *γ*2 and *γ*3, which were barely detected in the traditional method, and the discs large homologs family members, PSD‐95 and SAP97, which play important roles in trafficking and plasticity as postsynaptic scaffolding proteins (Cai et al., 2002; Fukata et al., 2005; Leonard et al., 1998; Schnell et al., 2002), an *α*/*β*‐hydrolase domain‐containing 12 protein (ABHD12), a known constituent of nAMPAR trafficking complexes (Schwenk et al., 2012), and Shisa 9, which is involved in AMPAR desensitization and short‐term plasticity at hippocampal synapses (von Engelhardt et al., 2010), were detected only in the rapidly isolated nAMPARs. Additionally, proteins involved in trafficking and recycling of nAMPARs, including N‐ethylmaleimide‐sensitive factor (NSF) (Song et al., 1998) and components of adaptor protein complex 2 (AP‐2) (Lee et al., 2002), were identified only in the rapid solubilization approach. Neuronal pentraxin 1, a known binding partner of ATD of AMPAR (Sia et al., 2007; Xu et al., 2003), was also co‐purified by the rapid method.

We were able to clearly observe the difference in the protein compositions from two methods after normalization of peptide counts (Figure 5b). nAMPARs solubilized for an extended period from crude membranes were more enriched with ER‐localized AMPAR assembly and trafficking partners such as ABHD6, CPT1C, FRRS1L, and SynDIG4, whereas nAMPAR rapidly solubilized from whole brain tissue retained interactions with a wider variety of binding proteins, from those involved in assembly and trafficking to synaptic localization.

### 2.4 Structural characterization of rapidly purified nAMPARs by cryo‐EM

**FIGURE 6:**
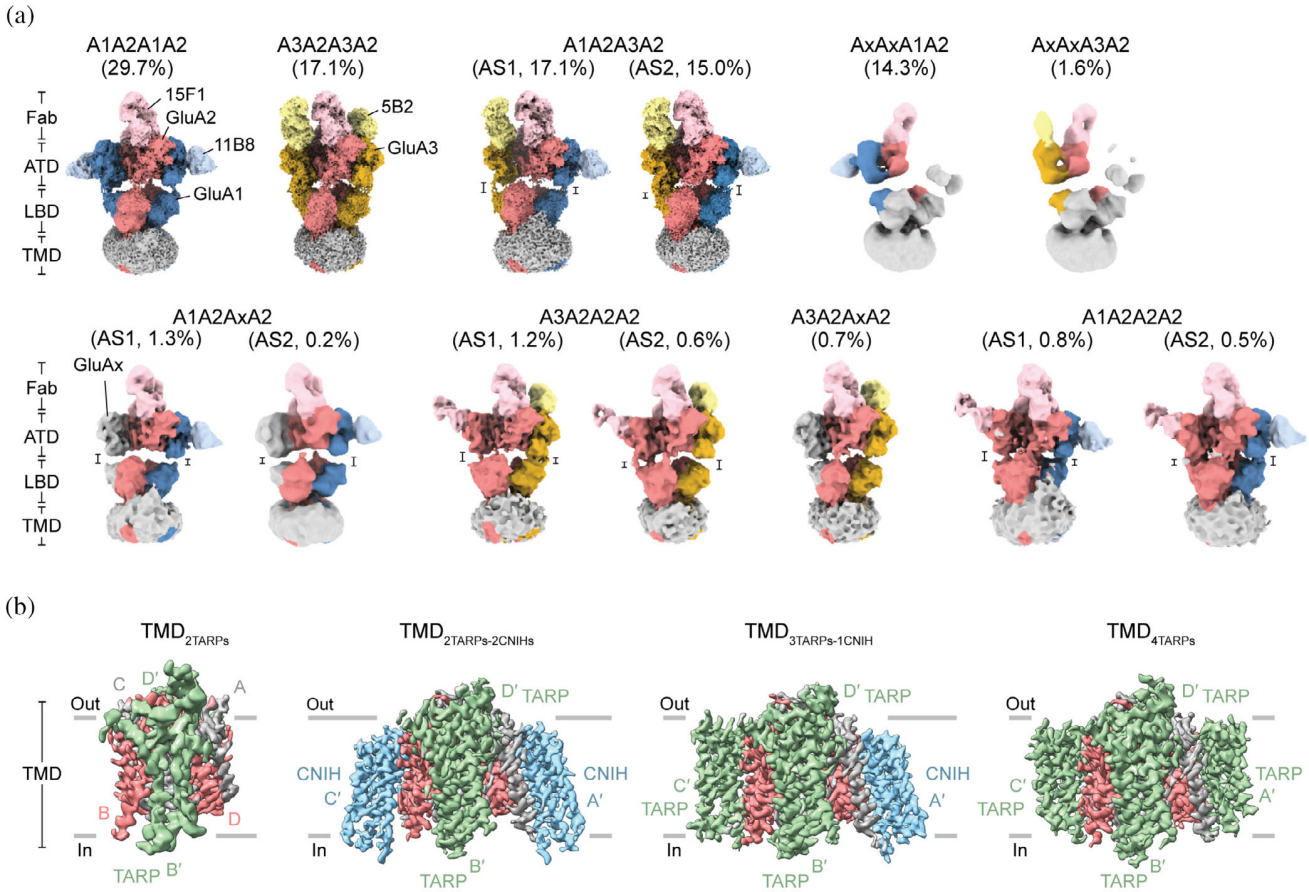
Subunit arrangements of rapidly purified nAMPARs. (a) Cryo‐EM maps of the 13 resolved subunit compositions of rapidly purified nAMPARs, classified by antibody fragment labeling of the ATD layer and symmetry. Ambiguous subunits (Ax) with no antibody fragments are shown in gray. (b) 3D reconstructions of TMD layer with four different auxiliary subunit compositions, viewed parallel to the membrane. TMD of the A/C and B/D positions are shown in gray and coral, respectively. TARPs and CNIHs are shown in green and cyan, respectively.

We further assessed the quality of rapidly purified nAMPARs by single‐particle cryo‐EM analysis. Initial 2D classification revealed that most receptor particles contain a stable ATD, LBD, and TMD layer with densities for 11B8 scFv, 15F1 Fab, and 5B2 Fab bound at the ATD layer (Figure S1). However, during initial heterogeneous refinement, we observed a particle class with a distinctive asymmetry in the ATD layer; one ATD dimer remained stable with well‐defined density, while the opposing dimer exhibited significantly attenuated and blurry density. 2D averages of this particle class clearly showed the half‐splayed ATD layer, indicating pronounced conformational flexibility of the poorly resolved ATD dimer (Figure S1b). Multiple rounds of 3D classification focused on the ATD layer with antibody fragments, followed by refinement, revealed seven distinct nAMPAR subunit compositions with different asymmetric “Y‐shaped” conformations, as well as two half‐splayed classes in which GluA1 or GluA3 occupies the C position and GluA2 is consistently in the D position (Figure 6a).

The populations of the various receptor complexes were consistent with previously determined subunit compositions of nAMPARs purified by membrane fraction of rat whole brain (Zhao et al., 2019). The triheteromeric A1A2A3A2 complex was predominant, accounting for 32% of particles, with diheteromeric A1A2A1A2 present at 30%, followed by diheteromeric A3A2A3A2 at 17% (Figures 6a and S2). The half‐splayed nAMPAR complexes, not reported in previous nAMPAR cryo‐EM analyses (Yu et al., 2021; Zhao et al., 2019), comprised a substantial fraction (17%) of particles. The ambiguous antibody labeling of the unstable ATD dimer, together with the low‐resolution reconstruction at the LBD–TMD layer, obscured subunit assignment, so these subunits were designated as Ax. Nontagged subunits in other classes were also assigned as Ax. Previous studies have shown that when GluA1 subunits occupy the B or D positions, the corresponding ATD dimer exhibits conformational flexibility (Fang et al., 2025; Zhang, Ivica, et al., 2023). Based on the pronounced flexibility of the half‐splayed particles, similar to A1 homotetramers (Zhang, Ivica, et al., 2023) or A1‐containing calcium‐permeable (CP) AMPARs (Fang et al., 2025), we speculate that the subunit in the B position in these classes is likely GluA1. In all classes, the D position was consistently occupied by GluA2 and the B position was also predominantly assigned as GluA2, in agreement with previous structural studies (Yu et al., 2021; Zhao et al., 2019). Notably, our cryo‐EM data did not uncover the A2A2A2A2, A2AxA1A2, and A2AxA3A2 subtypes, which were reported in a previous study (Zhao et al., 2019). The absence of these subtypes is likely due to their low particle abundance, preventing reliable identification and reconstruction.

We next evaluated the cryo‐EM data to uncover nAMPAR assemblies with auxiliary subunits or transiently binding partners, as suggested might be present by the mass spectrometry data (Figure 5b), using LBD–TMD or TMD focused 3D classification and refinement, because most of these partners—including ABHD12, TARP γ2/γ3, and Shisa 9—are transmembrane proteins. All particles with clear LBD‐TMD features were incorporated, given the high sequence conservation among GluA1 to GluA4, and identified into four distinct classes based on auxiliary subunit arrangements at the TMD layer (Figures 6b and S1, Table S1). The first and largest class with 281,775 particles (nominal resolution 3.55 Å, Figures 6b and S3a) exhibited two auxiliary subunits with distinct extracellular protrusions at the B′ and D′ positions and no density features at the A′ and C′ positions. Based on cryo‐EM density features with extracellular protrusions and transmembrane helices, we hypothesize that auxiliary subunits at the B′ and D′ positions in this class are TARP and are designated as TMD_2TARPs_ (Figure S3i). We were unable to specify the subtype of TARP in this class at this resolution. The second class, with a 2.87 Å resolution map focused on TMD (Figures 6b and S3b), revealed four auxiliary subunits at the A′, B′, C′, and D′ positions, where the B′ and D′ positions have densities of extracellular protrusions and distinct features of transmembrane helices, supporting their assignment as TARPs (Figure S3j). In contrast, no extracellular protrusions observed and distinct topology and feature of transmembrane helices at the A′ and C′ positions permitted us to assign CNIH2/3 to these densities and refer to this class as TMD_2TARPs‐2CNIHs_ (Figure S3j). Although CNIH2/3 were not detected in our mass spectrometry analysis (Figure 5b), likely due to their hydrophobicity and short extracellular and cytosolic loops, which limit the generation of detectable peptides during sample preparation for mass spectrometry, their characteristic structural features and density fit allowed for unambiguous assignment. The third class with a 3.00 Å focused TMD map (Figures 6b and S3c) exhibited noticeable differences in extracellular protrusions and transmembrane helices between the A′ and C′ positions. An auxiliary subunit at the A′ position displayed no extracellular protrusion and transmembrane helices as CNIH2/3 while densities at the C′ position exhibited extracellular protrusions, similar to those at the B′ and D′ positions, assigned as TARP (Figure S3k). We designated this class as TMD_3TARPs‐1CNIH_. The last class (2.99 Å, Figures 6b and S3d) contains four auxiliary subunits which all exhibited discernible extracellular protrusions, thus termed TMD_4TARPs_ (Figure S3l). Extracellular protrusions and four transmembrane helices of TARP at the B′ and D′ positions of TMD_2TARPs‐2CNIHs_, TMD_3TARPs‐1CNIH_ and TMD_4TARPs_ are well resolved with having distinct densities for TARP *γ*8‐specific negative allosteric modulator JNJ‐55511118 (JNJ, Figure S3j–l), supporting their assignment as TARP *γ*8. However, the densities of TARP at the C′ position of TMD_3TARPs‐1CNIH_ and the A′ and C′ positions of TMD_4TARPs_ are relatively weak and less resolved probably due to spatial constraints imposed by LBD, thus, we could not specify TARP subtype (Figure S3k, l).

The TMD_2TARPs‐2CNIHs_ and TMD_3TARPs‐1CNIH_ maps showed additional density features in a crevice between the M4 helix of the A and/or C positions and the TM4 helix of CNIH2/3 at the A′ and/or C′ positions, which could not be attributed to lipids or other small molecules (Figure S3m). Based on the spatial similarity to previous observation in the cryo‐EM of hippocampal nAMPARs (Yu et al., 2021) and mass spectrometry analysis (Figure 5b), we suggest that these densities are putative SynDIG4, which is known to regulate AMPAR properties and is correlated with the presence of CNIH2 (Martin et al., 2021; Matt et al., 2018; Schwenk et al., 2014). In addition, we observed spherical non‐protein densities at the center of the pore in the selectivity filter, which probably correspond to ions, such as sodium, or water molecules (Figure S3j–l). Lastly, comparison of core subunit compositions of four classes (Figure S3n) showed that in the TMD_2TARPs‐2CNIHs_ class, A1A2A1A2 receptors account for a larger population (38%) than other classes, which is in line with the preferred subunit arrangement of A1A2A1A2 with 2TARPs‐2CNIHs in hippocampal nAMPARs (Yu et al., 2021). On the other hand, the TMD_4TARPs_ class showed a slightly lower population (27%) of A1A2A1A2 receptors and a larger population (22%) of A3A2A3A2 receptors.

## 3 DISCUSSION

Isolation of native proteins from brain tissue is a powerful method to understand protein composition and structure, and to capture various interacting partners. However, retention of transient protein complexes during purification from native sources is challenging due to extended membrane preparation and purification. By using AMPARs, which are the most abundant ionotropic glutamatergic receptors, we demonstrate that modified strategies that solubilize and purify nAMPARs enable preparation of purified proteins within 2 h, which is 15 times faster than the traditional process. At the same time, utilizing whole brain tissue shows a higher yield, thus requiring less tissue. In addition, mass spectrometry analysis of rapidly purified nAMPARs reveals that more interacting proteins throughout the cell are co‐purified along with nAMPARs, and purified nAMPARs are suitable for high‐resolution cryo‐EM analysis. During cryo‐EM analysis, we identified distinct subunit compositions with a half‐splayed ATD layer. These classes, representing a substantial population, were not observed in previous nAMPAR studies that employed prolonged membrane solubilization (Yu et al., 2021; Zhao et al., 2019). We propose that our modified rapid isolation strategy was pivotal in preserving these states, as it captures a more comprehensive interactome across the cell, while previous methods potentially lose more metastable complexes. The asymmetric flexibility of the ATD layer in these receptors may provide the structural plasticity required for the receptor complexes to accommodate various macromolecular environments at the synapse. This conformational versatility could thus potentially offer a structural basis for the dynamic regulation of AMPAR complexes in different cellular contexts.

In addition, we observed four different auxiliary subunit compositions at the TMD, including a previously unreported arrangement of 3 TARPs and 1 CNIH, with a resolution high enough to detect putative ion densities in the central pore. Three auxiliary subunit constellations among these classes, 2TARPs, 2TARPs‐2CNIHs, and 4 TARPs, were also discovered in the CP‐AMPAR complexes, which adopted a modified rapid isolation method (Fang et al., 2025). Rapidly isolated CP‐AMPARs are also complexed with noelin, which is an olfactomedin domain‐containing secreted glycoprotein and known as an interacting protein to AMPARs for the synapse formation and receptor anchoring (Fang et al., 2025; Pandya et al., 2018; Watson et al., 2017). In this report, however, we are unable to reconstruct AMPARs complexed with many of the interacting proteins detected in the mass spectrometry. We infer that binding partners such as FRRS1L, SynDIG4 and Shisa 9 are sparsely populated given the low unique spectral counts, thus unable to be reliably identified and reconstructed. Highly populated interacting proteins such as ABHD6, NSF, CPT1C, PSD‐95, and AP‐2 likely bind to the unstructured C‐terminal domain of AMPARs in the cytosol given their predicted topologies and biochemical properties (Casas et al., 2020; Lee et al., 2002; Nishimune et al., 1998; Osten et al., 1998; Schnell et al., 2002; Song et al., 1998; Wei et al., 2016; Wei et al., 2017), which is challenging for 3D reconstruction. Further optimization and complementary strategy in cryo‐EM data processing may be required to visualize these complexes. Another possibility for failed reconstruction is that due to weak interaction with AMPARs, they potentially dissociate from AMPARs during cryo‐EM grid blotting and vitrification.

AMPARs exhibit extensive molecular and functional variability across neuronal cell types and brain regions (Geiger et al., 1995; Schwenk et al., 2014), as well as dynamic complex assemblies within different subcellular compartments during biogenesis, trafficking, and recycling (Matthews et al., 2021). In this study, whole brain tissues were utilized to establish a rapid isolation strategy. However, this approach inherently samples heterogeneous AMPAR complex compositions, which may limit the resolution of spatial information regarding AMPAR compositions and functions. Given that the narrower scope, such as focusing on specific brain regions or a subset of receptors, was helpful to elucidate region‐specific AMPAR compositions and previously unreported AMPAR complexes, as shown in hippocampal nAMPARs (Yu et al., 2021) and CP‐AMPARs from rat cerebellum (Fang et al., 2025), further strategies, such as employing subcellular fractionation or in vivo cross‐linking (Stark, 2010) to stabilize specific transient complexes, may be required.

The rapid solubilization and purification strategy presented here is a simple and efficient method to isolate complex and dynamic nAMPARs with their endogenous assemblies. Given an emergent interest into structural studies of membrane proteins from native sources (Jeong et al., 2022; Lin et al., 2021; Sun et al., 2023; Yang et al., 2023; Zhang et al., 2025; Zhao et al., 2019; Zhou et al., 2025; Zhu & Gouaux, 2021), this approach can be extended to other endogenous membrane proteins which form transient complexes, enabling their structural and functional study with minimal disturbance. Therefore, we anticipate this purification workflow of native membrane protein will serve as a versatile tool to facilitate the investigation of diverse macromolecular complexes of native membrane proteins.

## 4 METHOD

### 4.1 Expression and purification of mCherry‐3C‐3 × FLAG tagged anti‐GluA2 15F1 Fab

The anti‐GluA2 15F1 Fab construct was modified from a previous construct (Zhao et al., 2019). DNA sequences encoding the light and heavy chains of Fab domains were cloned into a bicistronic pFastBac1 vector for baculovirus‐mediated expression in Sf9 insect cells. Both light and heavy chains were fused at N‐termini with the GP64 signal peptide to facilitate secretion. For fluorescence‐based analysis and rapid immunoaffinity purification, the C‐terminus of the heavy chain was engineered to include a mCherry fluorescent protein tag, followed sequentially by an HRV 3C protease recognition site, a 3 × FLAG tag, a TEV protease cleavage site, and an octa‐histidine (His_8_) tag. Insect cells were transduced with baculovirus and cultured at 20°C. After 96 h, 2 L of the culture medium was harvested by centrifugation at 6400 *g* for 15 min, and the pH adjusted to 8.0. The supernatant was further clarified by centrifugation, filtered through a 0.22 μm membrane, and then concentrated to ~200 mL using tangential flow filtration with a 30‐kDa molecular‐weight cut‐off filter. The concentrated supernatant was dialyzed twice against a TBS buffer (20 mM Tris pH 8.0, 150 mM NaCl) for 24 h and then once more for 12 h against a TBS buffer supplemented with 10 mM imidazole. His‐tag affinity chromatography was performed to isolate 15F1 Fab initially, followed by the removal of His_8_ tag using TEV proteases during dialysis overnight against TBS buffer at 4°C. Cleaved tags and non‐cleaved proteins were removed by a second round of His‐tag affinity chromatography. His‐tag cleaved 15F1 Fab was further purified by anti‐FLAG affinity chromatography using anti‐FLAG magnetic agarose beads (Thermo Fisher Scientific, A36798) with elution using 2 × FLAG peptide (synthesized by GenScript), followed by SEC using a Superose 6 increase 10/300 GL column pre‐equilibrated with TBS. Peak fractions corresponding to 15F1 Fab were pooled and stored at −80°C.

### 4.2 Screening for optimal solubilization time of whole brain tissues

A single brain from a 7–8 week‐old C57BL/6 mouse, irrespective of sex, was dissected, immediately placed in, and washed with ice‐cold TBS buffer. A brain was homogenized using a Teflon‐glass homogenizer with 12 strokes in 3 mL of TBS buffer supplemented with 0.8 μM aprotinin, 2 μg mL^−1^ leupeptin, 2 μM pepstatin A, 1 mM phenylmethylsulfonyl fluoride (PMSF), 2 μM MPQX ([[3,4‐dihydro‐7‐(4‐morpholinyl)‐2,3‐dioxo‐6‐(trifluoromethyl)‐1(2*H*)‐quinoxalinyl]methyl]phosphonic acid), and 2 μM JNJ‐55511118 (5‐[2‐chloro‐6‐(trifluoromethoxy)phenyl]‐1,3‐dihydro‐2*H*‐benzimidazol‐2‐one). Homogenized whole brain tissues were immediately incubated with 3 mL of a homogenizing buffer supplemented with 4% (w/v) digitonin and excess amount, 20 μg, of 15F1 Fab‐mCherry‐3C‐3 × FLAG with stirring at 4°C. At designated time points (15 min, 30 min, 1 h, 2 h, 3 h, and 5 h), samples were taken, centrifuged at 4000 *g* for 5 min, and filtered through a 0.45 μm syringe filter. The resulting solubilized supernatants were analyzed by FSEC using a UFLC instrument (Shimadzu) equipped with a Superose 6 increase 10/300 GL column. Chromatography was performed at a flow rate of 0.5 mL min^−1^ in a FSEC buffer (20 mM Tris pH 8.0, 150 mM NaCl, 0.075% [w/v] digitonin, 2 μM MPQX, 2 μM (*R*, *R*)‐2b, and 2 μM JNJ‐55511118) and mCherry fluorescence was monitored with an excitation wavelength of 580 nm and an emission wavelength of 610 nm.

To compare the amount of insoluble nAMPARs remaining in the pellet after solubilization, pellets collected by centrifugation after 15 min or 3 h incubation were briefly washed with FSEC buffer and re‐centrifuged at 4000 *g* for 5 min. The insoluble pellets were then resuspended in TBS buffer supplemented with 1% (v/v) Triton X‐100, 0.5% (w/v) sodium deoxycholate, and incubated for 3 h with stirring at 4°C. The resulting re‐solubilized supernatants were clarified by centrifugation at 4000 *g* for 5 min, filtered, and analyzed by FSEC under the same conditions as described above.

### 4.3 Purification of anti‐GluA1 11B8 scFv and anti‐GluA3 5B2 Fab

Purification of the anti‐GluA1 11B8 scFv and anti‐GluA3 5B2 Fab was carried out as previously described (Yu et al., 2021).

### 4.4 Rapid solubilization and purification of nAMPARs from whole brain tissues

The supernatant from whole brain tissues of three mice solubilized briefly for 15 min was prepared as described above. To isolate nAMPARs using 15F1 Fab‐mCherry‐3C‐3 × FLAG, the solubilized supernatant was applied to 600 μL of anti‐FLAG magnetic agarose resin and incubated for 10 min on a rotator at 4°C. Magnetic resin was collected by placing the tube on a magnetic stand (Permagen, MSR1550), followed by careful removal of the supernatant. The resin was washed 3 times with a 5‐fold resin volume of FSEC buffer per wash. Bound nAMPAR‐15F1 Fab‐mCherry complexes were eluted by 5 min incubation with HRV 3C protease at a 5:1 weight ratio relative to 15F1 Fab in 1.5‐fold resin volume of FSEC buffer at 4°C. The resin was washed with 1.5‐fold resin volume of FSEC buffer and the wash was combined with protease‐eluted sample. To analyze the amount of nAMPARs remaining on the resin after protease elution, the resin was further incubated with 1 mg mL^−1^ of 2 × FLAG peptide in 1.5‐fold resin volume of FSEC buffer for 10 min at room temperature. The resin was washed again with 1.5‐fold resin volume of FSEC buffer and the wash was combined with peptide‐eluted sample. For the structural analysis, the protease‐eluted sample was incubated with an excess of 11B8 scFv and 5B2 Fab for 20 min at 4°C and subsequently loaded onto a Superose 6 increase 10/300 GL column connected to a UFLC instrument (Shimadzu). The column was equilibrated in a FSEC buffer and the protein elution was monitored by tryptophan fluorescence (excitation wavelength of 280 nm and emission wavelength of 335 nm) and mCherry fluorescence. The peak fractions were collected in two separate halves with the front half of the peak concentrated using a 100‐kDa MWCO concentrator for mass spectrometry and cryo‐EM grid preparation.

For comparison with nAMPARs purified via prolonged solubilization of crude membrane, crude membrane was prepared from the same mice used for rapid solubilization and purification, following previously described methods (Yu et al., 2021). Briefly, homogenized whole brain tissues were further disrupted by sonication for 90 s with 10 s on and 30 s off at medium power on ice. Intact cells and debris were removed by centrifugation at 4000 *g* for 5 min, and the membrane fraction was collected by ultracentrifugation at 200,000 *g* for 1 h at 4°C. Membranes were then solubilized in TBS buffer containing 2% (w/v) digitonin, protease inhibitors, allosteric modulators, and excess 15F1 Fab‐mCherry‐3C‐3 × FLAG, as described above, for 3 h with stirring at 4°C. The resulting supernatant was clarified by ultracentrifugation and filtration through a 0.45 μm syringe filter. Purification of nAMPARs from crude membrane was carried out as described above.

### 4.5 Mass spectrometry

The FSEC‐purified nAMPAR complexes from mouse brain were dried, dissolved in 5% sodium dodecyl sulfate, 8 M urea, 100 mM glycine (pH 7.55), reduced with tris(2‐carboxyethyl)phosphine at 37°C for 15 min, alkylated with methyl methanethiosulfonate for 15 min at room temperature followed by addition of acidified 90% methanol and 100 mM triethylammonium bicarbonate buffer (TEAB; pH 7.55). The sample was then digested in an S‐trap micro column briefly with 2 μg of a Tryp/LysC protease mixture, followed by a wash and 2 h digestion at 47°C with trypsin. The peptides were eluted with 50 mM TEAB and 50% acetonitrile, 0.2% formic acid, pooled and dried. Each sample was dissolved in 20 μL of 5% formic acid and injected into a Thermo Fisher QExactive HF mass spectrometer. Protein digests were separated using liquid chromatography with a Dionex RSLC UHPLC system, then delivered to a QExactive HF (Thermo Fisher) using electrospray ionization with a Nano Flex Ion Spray Source (Thermo Fisher) fitted with a 20 μm stainless steel nano‐bore emitter spray tip and 1.0 kV source voltage. Xcalibur version 4.0 was used to control the system. Samples were applied at 10 μL min^−1^ to a Symmetry C18 trap cartridge (Waters) for 10 min, then switched onto a 75 μm × 250 mm NanoAcquity BEH 130 C18 column with 1.7 μm particles (Waters) using mobile phases water (A) and acetonitrile (B) containing 0.1% formic acid, 7.5–30% acetonitrile gradient over 60 min and 300 nL min^−1^ flow rate. Survey mass spectra were acquired over *m/z* 375–1400 at 120,000 resolution (*m/z* 200) and data‐dependent acquisition selected the top 10 most abundant precursor ions for tandem mass spectrometry by higher energy collisional dissociation using an isolation width of 1.2 *m/z*, normalized collision energy of 30 and a resolution of 30,000. Dynamic exclusion was set to auto, charge state for MS/MS +2 to +7, maximum ion time 100 ms, minimum AGC target of 3 × 10^6^ in MS1 mode and 5 × 10^3^ in MS2 mode. Data analysis was performed using Comet (v. 2016.01, rev. 3) (Eng et al., 2013) against a September 2021 version of canonical FASTA protein database containing *Mus musculus* UniProt sequences and concatenated sequence‐reversed entries to estimate error thresholds and 179 common contaminant sequences and their reversed forms. Comet searches for all samples performed with trypsin enzyme specificity with monoisotopic parent ion mass tolerance set to 1.25 Da and monoisotopic fragment ion mass tolerance set at 1.0005 Da. A static modification of +45.9877 Da was added to all cysteine residues and a variable modification of +15.9949 Da on methionine residues. A linear discriminant transformation was used to improve the identification sensitivity from the Comet analysis (Keller et al., 2002; Wilmarth et al., 2009). Separate histograms were created for matches to forward sequences and for matches to reversed sequences for all peptides of seven amino acids or longer. The score histograms of reversed matches were used to estimate peptide false discovery rates (FDR) and set score thresholds for each peptide class. The overall protein FDR was 1.3%.

### 4.6 Cryo‐EM sample preparation and data acquisition

A 2.5 μL aliquot of nAMPARs from the front half of the peak at a concentration of 0.12 mg mL^−1^, as determined by OD_280_, was applied to a Quantifoil R2/1200 mesh gold grid covered by 2 nm continuous carbon film, which was glow‐discharged in the presence of amylamine for 30 s at 15 mA. The grids were blotted using a Vitrobot mark IV for 2.5 s with a blot force of 0 after 30 s wait time under 100% humidity at 15°C. The grids were flash‐frozen into liquid ethane, cooled by liquid nitrogen.

Cryo‐EM dataset was collected on a 300 keV Titan Krios microscope with a K3 detector and an energy filter set to a 20 eV slit width at a magnification of 105,000×, corresponding to a pixel size of 0.4195 Å per pixel in super‐resolution mode. Images were collected by a 3 × 3 multi‐hole per stage shift and a 6 multi‐shot per hole method using SerialEM, with a defocus range of −1.0 to −2.2 μm. Each movie stack was collected with 50 frames for a total exposure time of 3.73 s at a dose rate of ~6.7 e^−^ per pixel per s, resulting in a total dose of 50 e^−^ Å^−2^. A total of 33,133 movies were collected.

### 4.7 Cryo‐EM image processing

All cryo‐EM images were processed by cryoSPARC (v3.3.2–4.7.0). Beam‐induced motion was corrected by patch motion correction with an output Fourier cropping factor of 1/2 (0.839 Å per pixel). Contrast transfer function (CTF) parameters were estimated by patch CTF estimation. Initially, 5.8 million particles were picked by using blob picker with minimum and maximum particle diameters of 180 and 250 Å, respectively, extracted with a box size of 600 × 600 pixels and downsampled by 4× binning. Several rounds of 2D classification were carried out and particles with clear AMPAR features, approximately 1 million, were reconstructed using ab initio reconstruction (*N* = 3) and used as a template for the template picker later. The 1 million particle stack was re‐extracted to a box size of 600 × 600 pixels and downsampled by 2× binning and subjected to heterogeneous refinement using the reconstructed model from the ab initio reconstruction. Classes with clear receptor features with two 15F1 Fabs in the B/D positions and two 11B8 scFv in the A/C positions at the ATD layer (called A1A2A1A2) were selected and used for heterogeneous refinement with non‐clarified blob‐picked and 4 × binned 5.8 million particles, yielding 10 classes. Among them, one class of 891,491 particles with clear AMPAR features was subjected to one round of 2D classification to remove additional junk particles, resulting in 796,762 particles. In parallel to blob picked particles, another particle set from the entire dataset (33,133 movies) was picked using template‐based picker and extracted with a box size of 600 × 600 pixels and downsampled by 4× binning. After the inspection of picked particles, heterogeneous refinement was performed with the resulting 20 million template‐picked particles to sort out clear AMPAR particles, yielding 6 classes. 6 million particle classes with clear AMPAR features were subjected to two rounds of 2D classification to remove junk particles, resulting in 2,715,322 particles. Then, duplicates were removed from 796,762 particle set from blob picker and 2,715,322 particle set from template picker and several rounds of 2D classification were carried out again. Resulting 1,185,588 particles were reconstructed using ab initio reconstruction (*N* = 5) and classified into 8 classes by heterogeneous refinement. Among them, one class showing weaker A, B density was further classified by two rounds of 2D classification and 23,577 particles having half of ATD layer with clear ATD and Fab/scFv signal, but the other half of ATD layer with smear and splayed signal were selected and reconstructed by ab initio reconstruction (*N* = 3). A consensus map of the half‐splayed ATD layer was generated by homogeneous refinement and non‐uniform refinement and subsequently used as a reference with other classes for several rounds of heterogeneous refinement. These classifications separated the particles into 4 classes with different, but still heterogeneous antibody labeling: class 1 (splayed ATD layers in the A/B positions, 11B8 scFv in the C position, and 15F1 Fab in the D position), class 2 (two 15F1 Fab in the B/D positions, mixed 11B8 scFv and 5B2 Fab in the A position, and 5B2 Fab in the C position), class 3 (two 15F1 Fab in the B/D positions, 11B8 scFv in the A position, and mixed 5B2 Fab and 11B8 scFv in the C position), and class 4 (two 15F1 Fab in the B/D positions and two 11B8 scFv in the A/C positions). To achieve distinct separation of antibody labeling, each class was classified by iterative 3D classifications with ATD focused mask. From these, class 1 with half‐splayed ATD layer was sorted out into two classes: AxAxA1A2 and AxAxA3A2, where “x” represents the subunit that was unresolvable due to flexibility or not labeled by any of the antibody fragments. The other three classes were further classified by ATD focused 3D classification, which allowed separation of single antibody fragment labeling in each subunit and revealed minor classes that had previously been obscured due to their low particle number. Receptor classes bound with the same Fab/scFv combination and sharing the same asymmetric (AS) orientation of the ATD layer were combined. After non‐uniform refinement, 13 subtypes were resolved: A1A2A1A2, A3A2A3A2, A1A2A3A2 (AS1), A1A2A3A2 (AS2), AxAxA1A2, AxAxA3A2, A1A2AxA2 (AS1), A1A2AxA2 (AS2), A3A2A2A2 (AS1), A3A2A2A2 (AS2), A3A2AxA2, A1A2A2A2 (AS1), and A1A2A2A2 (AS2).

To identify the auxiliary subunits of the LBD–TMD layer, all particle classes displaying receptor features with antibody labeling in heterogeneous refinement (a) were combined and re‐extracted with a box size of 512 × 512 pixels, and a consensus map with heterogeneous antibody labeling was generated using non‐uniform refinement. An inverted mask outside the LBD–TMD layer was generated from this map and applied for particle subtraction to subtract the ATD layer and antibody fragments. Then, particles with incomplete subtraction, as well as junk particles, were sorted out by two rounds of 2D classification, resulting in 963,648 particles. A good reference was generated by ab initio reconstruction (*N* = 3), followed by homogeneous refinement and non‐uniform refinement. Subsequent 3D classification (*N* = 6) and non‐uniform refinement of each class reveal that 34.57% of particles displayed clear auxiliary subunit densities at all A′, B′, C′, and D′ positions, whereas 29.24% of particles with a 3.55 Å focused TMD map after TMD‐focused local refinement with C2 symmetry exhibited strong densities at the B′ and D′ positions corresponding to TARP, but no densities at the A′ and C′ positions. A class with full occupation at the A′, B′, C′, and D′ positions displayed weak extracellular protrusions at the A′ and C′ positions when viewed at a low contour level. Therefore, after TMD‐focused local refinement, an additional round of 3D classification (*N* = 4), focusing on the A′ and C′ positions, was performed, yielding two distinct particle classes: 24.73% of particles with a 3.00 Å map showed no extracellular protrusion at the A′ position, corresponding to CNIH2/3, and prominent extracellular protrusions at the B′, C′, and D′ positions, corresponding to TARP, whereas 26.66% of particles with a 2.87 Å map (C2) exhibited no extracellular protrusions at the A′ and C′ positions, corresponding to CNIH2/3. Another round of focused 3D classification of one class with extracellular protrusions at the A′ and C′ positions classified 51.21% of particles with a 2.99 Å map (C2) showing extracellular protrusions at all A′, B′, C′, and D′ positions, corresponding to TARP.

### 4.8 Model building

The initial EM density map was sharpened with Phenix AutoSharpen (Adams et al., 2002) and both unsharpened and sharpened maps were used for structural determination. The structural modeling of TMD with auxiliary subunits was carried out using rigid body fitting of the structure of LBD–TMD from all hippocampal nAMPAR subtypes (PDB ID: 7LEP) using UCSF Chimera (Pettersen et al., 2021; Yu et al., 2021), after removing the LBD. The auxiliary subunits were rigid‐body fitted independently into the cryo‐EM densities and prominent tube‐shaped electron densities surrounding TMD likely represent ordered lipid molecules as previously reported (Yu et al., 2021). Therefore, we modeled these densities using alkane chains of corresponding length and the structures were manually adjusted in Coot (Emsley et al., 2010). Subsequently, the models were refined by real‐space refinement using Phenix (Afonine et al., 2018).

## AUTHOR CONTRIBUTIONS

**Jumi Park:** Conceptualization; investigation; methodology; validation; data curation; formal analysis; visualization; writing – original draft; writing – review and editing. **Eric Gouaux:** Conceptualization; methodology; supervision; resources; funding acquisition; project administration; writing – original draft; writing – review and editing.

## CONFLICT OF INTEREST STATEMENT

The authors declare no competing interests.

## Supporting information


**Data S1:** Supporting Information

## Data Availability

The cryo‐EM maps for A1A2A1A2, A3A2A3A2, A1A2A3A2 (AS1), A1A2A3A2 (AS2), AxAxA1A2, AxAxA3A2, A1A2AxA2 (AS1), A1A2AxA2 (AS2), A3A2A2A2 (AS1), A3A2A2A2 (AS2), A3A2AxA2, A1A2A2A2 (AS1), A1A2A2A2 (AS2) and the TMD_2TARPs_ have been deposited in the Electron Microscopy Data Bank (EMDB) under accession code EMD‐73846, EMD‐73847, EMD‐73848, EMD‐73849, EMD‐73850, EMD‐73851, EMD‐73852, EMD‐73853, EMD‐73854, EMD‐73855, EMD‐73856, EMD‐73858, EMD‐73859 and EMD‐73860. The cryo‐EM maps and coordinates for the TMD_2TARPs‐2CNIHs_, TMD_3TARPs‐1CNIH_, and TMD_4TARPs_ have been deposited in the EMDB under accession codes EMD‐73861, EMD‐73862 and EMD‐73863 and in the Protein Data Bank (PDB) under accession codes 9Z6U, 9Z6V and 9Z6W, respectively.
