# Supplementary material for: Efficient and rapid isolation of native AMPA receptor complexes for cryo‐EM
Source: Protein Sci. 2026 Jan 24;35(2):e70483. doi: 10.1002/pro.70483 (PMC12831286; doi:10.1002/pro.70483)
Supplement: Supplementary file 1 — Data S1: Supporting Information [file PRO-35-e70483-s001.pdf]

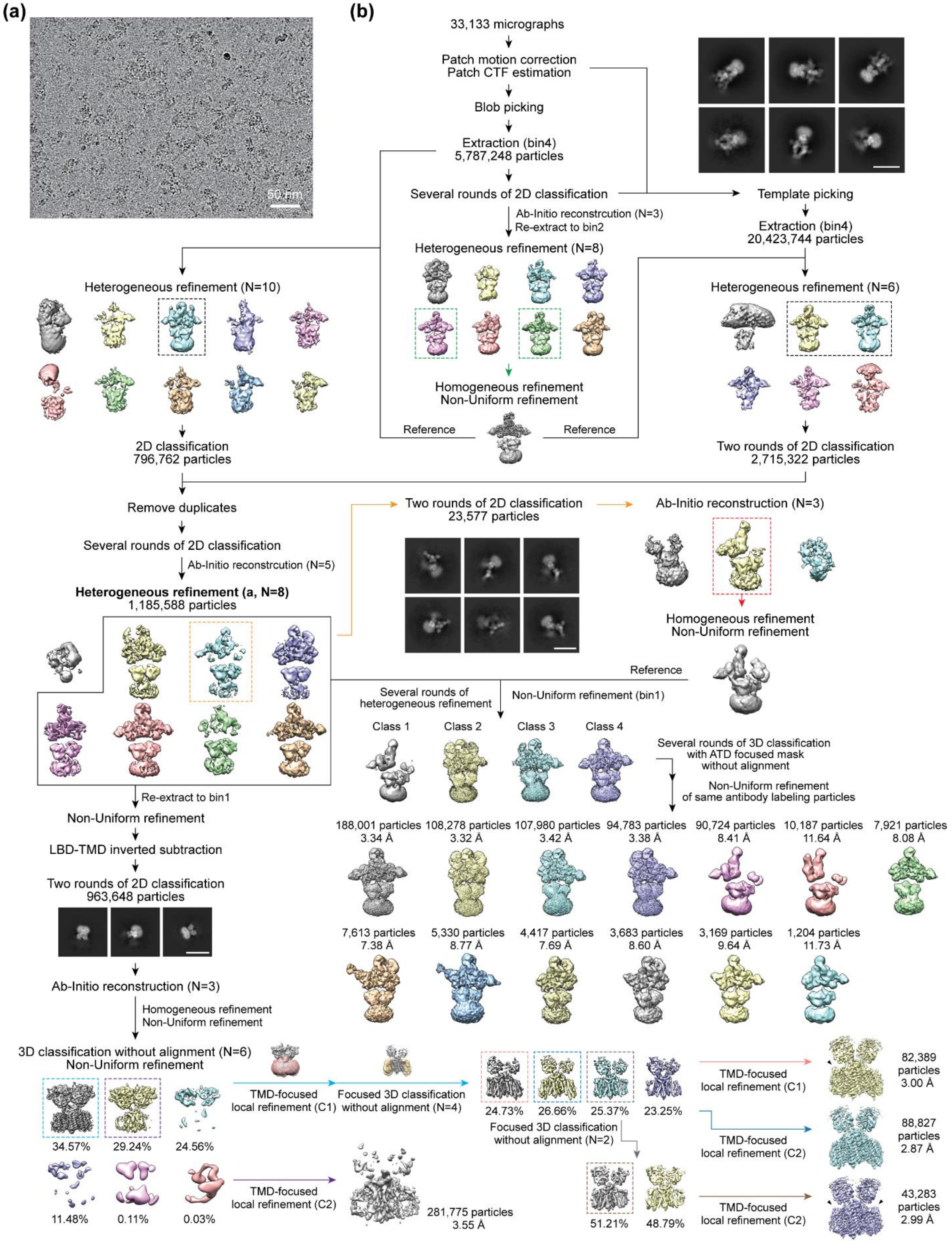
(a)
50 nm
(b)
33,133 micrographs
Patch motion correction
Patch CTF estimation
Blob picking
Extraction (bin4)
5,787,248 particles
Several rounds of 2D classification
Ab-Initio reconstrcution (N=3)
Re-extract to bin2
Heterogeneous refinement (N=8)
Homogeneous refinement
Non-Uniform refinement
Reference
Reference
Template picking
Extraction (bin4)
20,423,744 particles
Heterogeneous refinement (N=6)
Two rounds of 2D classification
2,715,322 particles
Heterogeneous refinement (N=10)
2D classification
796,762 particles
Remove duplicates
Several rounds of 2D classification
Ab-Initio reconstrcution (N=5)
Heterogeneous refinement (a, N=8)
1,185,588 particles
Two rounds of 2D classification
23,577 particles
Ab-Initio reconstruction (N=3)
Homogeneous refinement
Non-Uniform refinement
Reference
Several rounds of
heterogeneous refinement
Non-Uniform refinement (bin1)
Class 1
Class 2
Class 3
Class 4
Several rounds of 3D classification
with ATD focused mask
without alignment
Non-Uniform refinement
of same antibody labeling particles
188,001 particles
3.34 Å
108,278 particles
3.32 Å
107,980 particles
3.42 Å
94,783 particles
3.38 Å
90,724 particles
8.41 Å
10,187 particles
11.64 Å
7,921 particles
8.08 Å
7,613 particles
7.38 Å
5,330 particles
8.77 Å
4,417 particles
7.69 Å
3,683 particles
8.60 Å
3,169 particles
9.64 Å
1,204 particles
11.73 Å
Re-extract to bin1
Non-Uniform refinement
LBD-TMD inverted subtraction
Two rounds of 2D classification
963,648 particles
Ab-Initio reconstruction (N=3)
Homogeneous refinement
Non-Uniform refinement
3D classification without alignment (N=6)
Non-Uniform refinement
34.57%
29.24%
24.56%
11.48%
0.11%
0.03%
TMD-focused
local refinement (C1)
Focused 3D classification
without alignment (N=4)
24.73%
26.66%
25.37%
23.25%
Focused 3D classification
without alignment (N=2)
51.21%
48.79%
TMD-focused
local refinement (C2)
281,775 particles
3.55 Å
TMD-focused
local refinement (C1)
82,389
particles
3.00 Å
TMD-focused
local refinement (C2)
88,827
particles
2.87 Å
TMD-focused
local refinement (C2)
43,283
particles
2.99 Å

Figure S1. Cryo-EM data processing of rapidly purified nAMPARs.

(a) Representative motion-corrected cryo-EM micrograph of rapidly purified nAMPARs. **(b)** A flow chart of cryo-EM data processing. Scale bars in representative 2D averages indicate 200 Å. Black arrow heads in the bottom right corner show distinct extracellular protrusions of auxiliary subunits at the A′ and/or C′ positions.

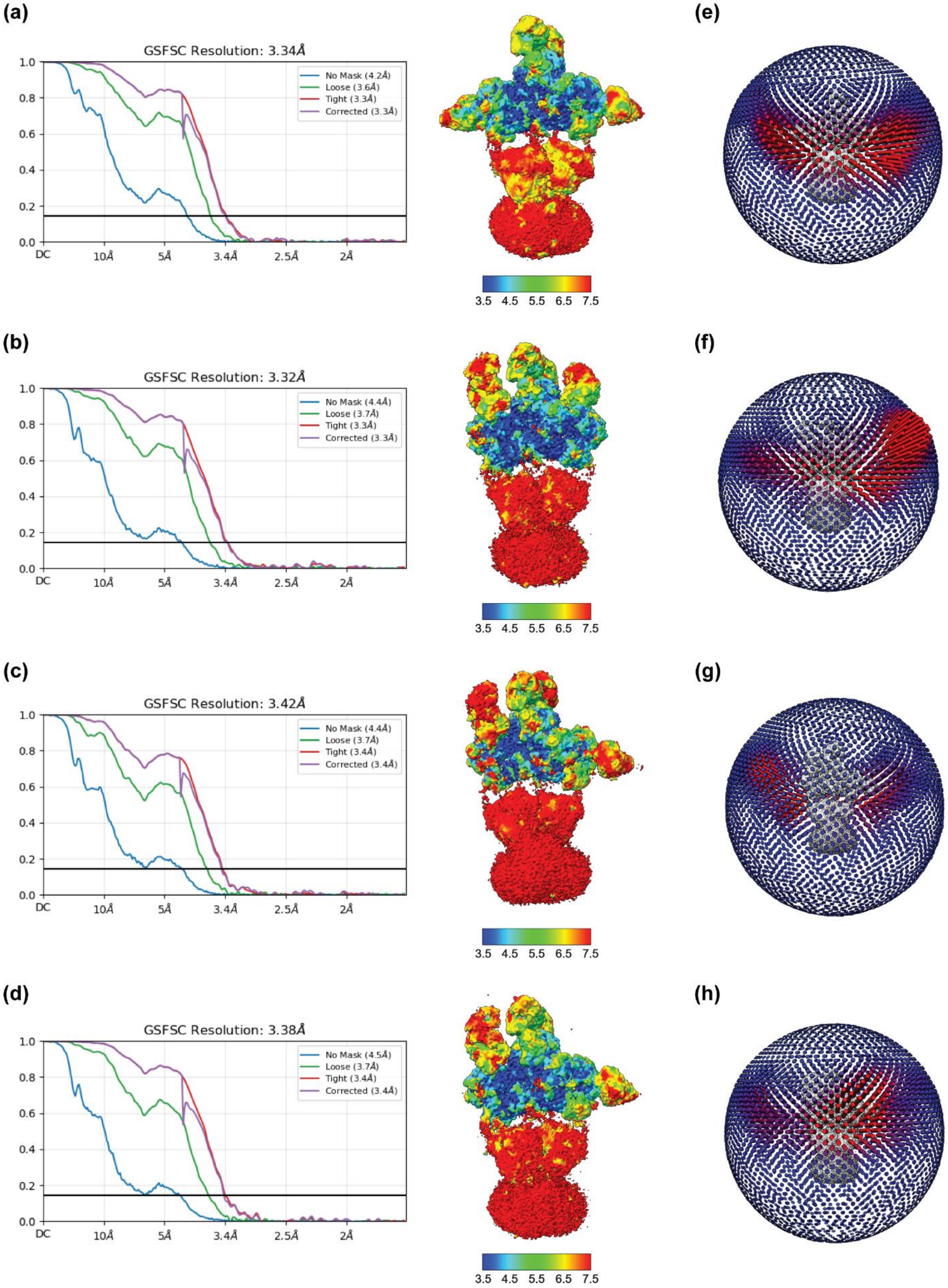
(a)
GSFSC Resolution: 3.34Å
No Mask (4.2Å)
Loose (3.6Å)
Tight (3.3Å)
Corrected (3.3Å)
1.0
0.8
0.6
0.4
0.2
0.0
DC
10Å
5Å
3.4Å
2.5Å
2Å
3.5 4.5 5.5 6.5 7.5
(e)
(b)
GSFSC Resolution: 3.32Å
No Mask (4.4Å)
Loose (3.7Å)
Tight (3.3Å)
Corrected (3.3Å)
3.5 4.5 5.5 6.5 7.5
(f)
(c)
GSFSC Resolution: 3.42Å
No Mask (4.4Å)
Loose (3.7Å)
Tight (3.4Å)
Corrected (3.4Å)
3.5 4.5 5.5 6.5 7.5
(g)
(d)
GSFSC Resolution: 3.38Å
No Mask (4.5Å)
Loose (3.7Å)
Tight (3.4Å)
Corrected (3.4Å)
3.5 4.5 5.5 6.5 7.5
(h)

Figure S2. Cryo-EM statistics and local density maps for nAMPARs.

(a-d) Fourier shell correlation (FSC) curves (left) and local resolution estimates (right) of A1A2A1A2 (a), A3A2A3A2 (b), A1A2A3A2 (AS1) (c) and A1A2A3A2 (AS2) (d). **(e-h)** Euler angle distribution of particles used for final cryo-EM reconstructions of A1A2A1A2 (e), A3A2A3A2 (f), A1A2A3A2 (AS1) (g) and A1A2A3A2 (AS2) (h).

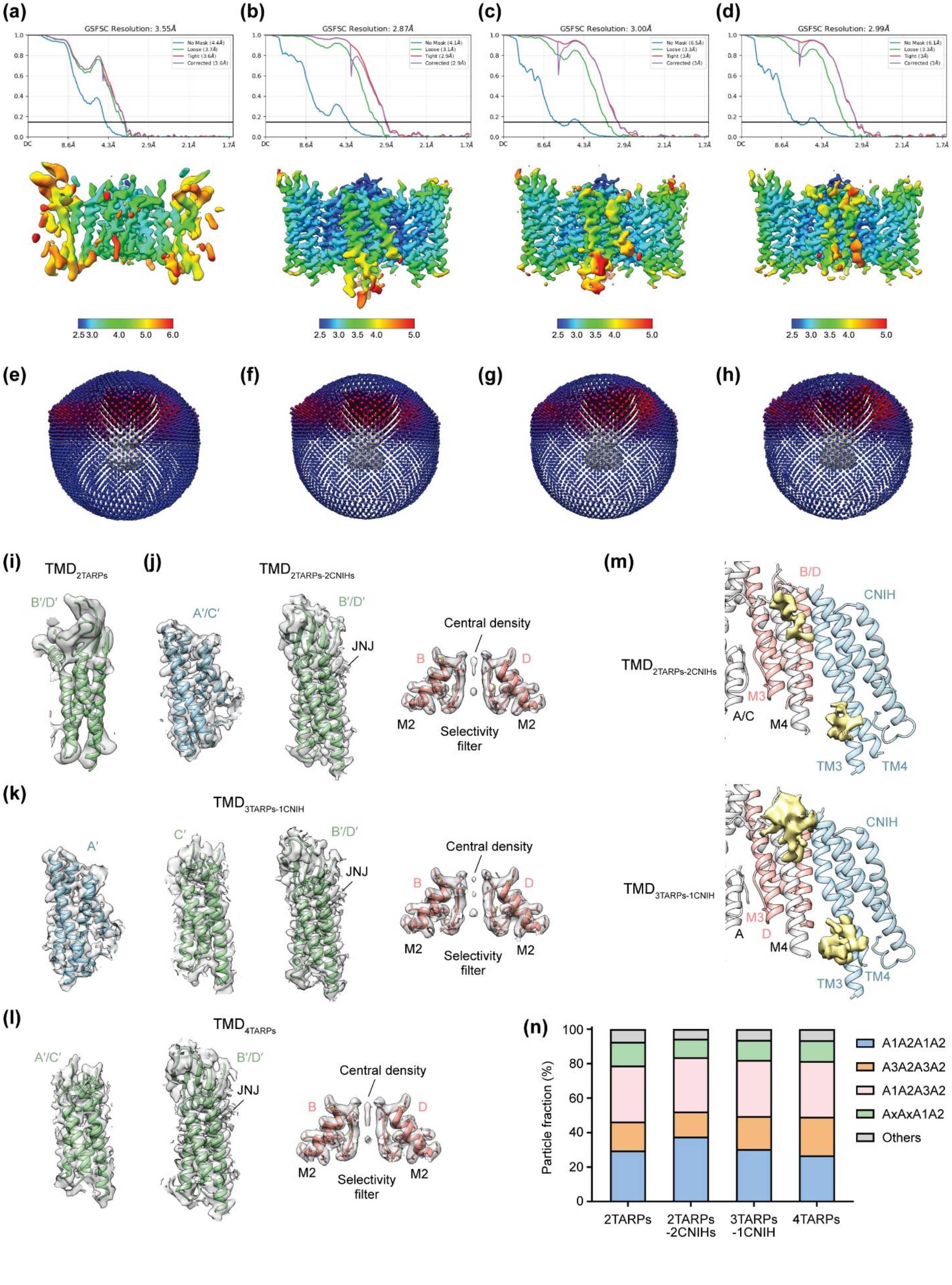

(a)
GSFSC Resolution: 3.55Å
(b)
GSFSC Resolution: 2.87Å
(c)
GSFSC Resolution: 3.00Å
(d)
GSFSC Resolution: 2.99Å
(e)
(f)
(g)
(h)
(i)
TMD2TARPs
B′/D′
(j)
TMD2TARPs-2CNIHs
A′/C′
B′/D′
JNJ
Central density
B
D
M2
M2
Selectivity filter
(m)
B/D
CNIH
TMD2TARPs-2CNIHs
M3
A/C
M4
TM3
TM4
(k)
TMD3TARPs-1CNIH
A′
C′
B′/D′
JNJ
Central density
B
D
M2
M2
Selectivity filter
CNIH
TMD3TARPs-1CNIH
M3
A
D
M4
TM3
TM4
(l)
TMD4TARPs
A′/C′
B′/D′
JNJ
Central density
B
D
M2
M2
Selectivity filter
(n)
Particle fraction (%)
A1A2A1A2
A3A2A3A2
A1A2A3A2
AxAxA1A2
Others
2TARPs
2TARPs -2CNIHs
3TARPs -1CNIH
4TARPs

Figure S3. Cryo-EM statistics and local density maps for TMD layers with different auxiliary subunit compositions and core subunit compositions of each class.

(a-d) Fourier shell correlation (FSC) curves (top) and local resolution estimates (bottom) of the TMD_{2TARPs} map (a), the $TMD_{2TARPs\text{-}2CNIHs}$ (b), the $TMD_{3TARPs\text{-}1CNIH}$ (c) and the TMD_{4TARPs} (d). **(e-h)** Euler angle distribution of particles used for final cryo-EM reconstructions of TMD_{2TARPs} (e), $TMD_{2TARPs\text{-}2CNIHs}$ (f), $TMD_{3TARPs\text{-}1CNIH}$ (g) and TMD_{4TARPs} (h). **(i)** The local map shows auxiliary subunit densities in the TMD_{2TARPs} map, contoured at 0.1σ. **(j)** The local maps show auxiliary subunit densities and the M2 helices and the selectivity filters in the $TMD_{2TARPs\text{-}2CNIHs}$ map, contoured at 0.1σ. **(k)** The local maps show auxiliary subunit densities and the M2 helices and the selectivity filters in the $TMD_{3TARPs\text{-}1CNIH}$ map, contoured at 0.09σ. **(l)** The local maps show auxiliary subunit densities and the M2 helices and the selectivity filters in the TMD_{4TARPs} map, contoured at 0.08σ. **(m)** The additional densities for putative SynDIG4 at a crevice between the M4 helix of the A/C positions and the TM4 helix of CNIH2/3 in the $TMD_{2TARPs\text{-}2CNIHs}$ map (top, contoured at 0.1σ) and the $TMD_{3TARPs\text{-}1CNIH}$ map (bottom, contoured at 0.09σ) are shown in yellow. **(n)** The graph shows the core subunit compositions of each auxiliary subunit arrangement in the TMD layer.

Table S1. Statistics of cryo-EM data collection, refinement and validation.

	Native AMPAR + MPQX, JNJ, *(R, R)*-2b	
**Data collection and processing**		
Magnification	105,000×	
Voltage (kV)	300	
Electron exposure (e–/Å^2)	50	
Defocus range (μm)	-1.0 to -2.2	
Pixel size (Å)	0.839	
	**ATD focused classification**	**TMD focused classification**
Initial particle images (no.)	1,068,075	963,648
Final particle images (no.)	188,001 (A1A2A1A2)<br>108,278 (A3A2A3A2)<br>107,980 (A1A2A3A2-AS1)<br>94,783 (A1A2A3A2-AS2)<br>90,724 (AxAxA1A2)<br>10,187 (AxAxA3A2)<br>7,921 (A1A2AxA2-AS1)<br>7,613 (A3A2A2A2-AS1)<br>5,330 (A1A2A2A2-AS1)<br>4,417 (A3A2AxA2)<br>3,683 (A3A2A2A2-AS2)<br>3,169 (A1A2A2A2-AS2)<br>1,204 (A1A2AxA2-AS2)	281,775 (TMD_{2TARPs})<br>88,827 ($TMD_{2TARPs-2CNIHs}$)<br>82,389 ($TMD_{3TARPs-1CNIH}$)<br>43,283 (TMD_{4TARPs})

	$TMD_{2TARPs-2CNIHs}$ (EMDB-73861) (PDB 9Z6U)	$TMD_{3TARPs-1CNIH}$ (EMDB-73862) (PDB 9Z6V)	TMD_{4TARPs} (EMDB-73863) (PDB 9Z6W)
**Refinement**			
Symmetry imposed	C2	C1	C2
Map resolution (Å)	2.87	3.00	2.99
FSC threshold	0.143	0.143	0.143
Map sharpening *B* factor (Å^2)	73.8	70.4	72.4
Model resolution (Å)	3.02	3.20	3.15
FSC threshold	0.5	0.5	0.5
Model composition			
Non-hydrogen atoms	9242	8797	8704
Protein residues	1156	1138	1144
Ligands	46	28	30
B factors (Å^2)			
Protein	67.44	61.42	63.45
Ligand	62.06	48.65	51.41
R.m.s. deviations			
Bond lengths (Å)	0.003	0.004	0.003
Bond angles (°)	0.492	0.501	0.506
Validation			
MolProbity score	1.36	1.51	1.47
Clashscore	4.84	5.35	5.09
Poor rotamers (%)	1.24	1.43	1.59
Ramachandran plot			
Favored (%)	97.83	97.51	97.79
Allowed (%)	2.17	2.49	2.21
Disallowed (%)	0.00	0.00	0.00
